# Losartan Decreases p42/44 MAPK Signaling and Preserves LZ+ MYPT1 Expression

**DOI:** 10.1371/journal.pone.0005144

**Published:** 2009-04-09

**Authors:** Erhan Ararat, Frank V. Brozovich

**Affiliations:** Division of Cardiovascular Diseases, Mayo Medical School, Rochester, Minnesota, United States of America; University of Cincinnati, United States of America

## Abstract

Heart failure is associated with impairment in nitric oxide (NO) mediated vasodilatation, which has been demonstrated to result from a reduction in the relative expression of the leucine zipper positive (LZ+) isoform of the myosin targeting subunit (MYPT1) of myosin light chain phosphatase. Further, captopril preserves normal LZ+ MYPT1 expression, the sensitivity to cGMP-mediated vasodilatation and modulates the expression of genes in the p42/44 MAPK and p38 MAPK signaling cascades. This study tests whether angiotensin receptor blockade (ARB) with losartan decreases p42/44 MAPK or p38 MAPK signaling and preserves LZ+ MYPT1 expression in a rat infarct model of heart failure. In aortic smooth muscle, p42/44 MAPK activation increases and LZ+ MYPT1 expression falls after LAD ligation. Losartan treatment decreases the activation of p42/44 MAPK to the uninfarcted control level and preserves normal LZ+ MYPT1 expression. The expression and activation of p38 MAPK, however, is low and does not change following LAD ligation or with losartan therapy. These data suggest that either reducing or blocking the effects of circulating angiotensin II, both decreases the activation of the p42/44 MAPK signaling cascade and preserves LZ+ MYPT1 expression. Thus, the ability of ACE-inhibitors and ARBs to modulate the vascular phenotype, to preserve normal flow mediated vasodilatation may explain the beneficial effects of these drugs compared to other forms of afterload reduction in the treatment of heart failure.

## Introduction

Activation of smooth muscle is dependent on the level of phosphorylation of the 20 kDa regulatory myosin light chain (MLC_20_), which is determined by the relative activities of MLC kinase (MLCK) and MLC phosphatase [1], [2]. Until recently, activation and relaxation of smooth muscle were thought to be regulated by MLCK, while MLC phosphatase was an unregulated housekeeping enzyme [3]. However, evidence indicates that the majority of signaling pathways for the regulation of vascular tone converge on MLC phosphatase [4], [5].

MLC phosphatase isolated from smooth muscle is a holoenzyme consisting of three subunits [4]; a 20 kDa subunit of unknown function, a 38 kDa catalytic subunit and a myosin targeting subunit (MYPT1) of 110–133 kDa. Alternative splicing of two different exons generates four MYPT1 isoforms. One exon codes for the presence or absence of a 41 aa central insert [5]. The other is a 31 bp 3′ exon; exon inclusion codes for a MYPT1 that lacks a COOH-terminus leucine zipper (LZ−), while exon exclusion shifts the reading frame and codes for a LZ+ MYPT1 isoform [6].

Nitric oxide (NO) is the classical agent to produce Ca^2+^ desensitization [7], and NO-mediated, or flow-mediated, vasodilatation is a fundamental response of the vasculature [8]. In the vasculature, an increase in flow increases shear stress on endothelial cells, which stimulates NO production. NO diffuses into the smooth muscle cells to activate the soluble pool of guanylate cyclase, to increase cGMP, which then activates type I cGMP-dependent protein kinase (PKGI), which subsequently produces smooth muscle relaxation by its interactions with the maxi K^+^ channel [9], the SR and voltage dependent Ca^2+^ channels [10], [11] and MLC phosphatase [12]. In addition, PKGI dependent pathways for vasodilatation include a phosphorylation of telokin [13], [14], [15] and HSP20 [16].

A number of groups have demonstrated that the sensitivity to cGMP-mediated smooth muscle cell relaxation correlates with the relative expression of LZ+/LZ− MYPT1 isoforms [6], [17], [18], [19], [20], suggesting that the relative expression of LZ+/LZ- MYPT1 isoforms could determine the sensitivity of the smooth muscle to NO mediated vasodilatation [12]. However, not only does the relative expression of LZ+/LZ− MYPT1 isoforms correlate with the sensitivity of cGMP-mediated relaxation; we have demonstrated that changes in LZ+/LZ− MYPT1 expression, in isolation, cause changes in the sensitivity to cGMP-mediated smooth muscle relaxation [21].

We have previously demonstrated that between 2–4 weeks following a myocardial infarction, the expression of the LZ+ MYPT1 isoform decreases and this is accompanied by a decrease in the sensitivity to NO mediated vasodilatation [22]. Further captopril therapy preserves both normal LZ+ MYPT1 expression and sensitivity to NO mediated vasodilatation [22] as well as suppresses the expression of genes involved in p42/44 MAPK and p38 MAPK signaling [23]. The present study examined whether captopril treatment of heart failure maintains LZ+ MYPT1 expression by decreasing angiotensin II (Ang II) by testing the ability of angiotensin receptor blocker (ARB) therapy to maintain LZ+ MYPT1 expression and decrease the activation of either p42/44 or p38 MAPK.

## Results

### Left Ventricular Function

**Figure 1:**
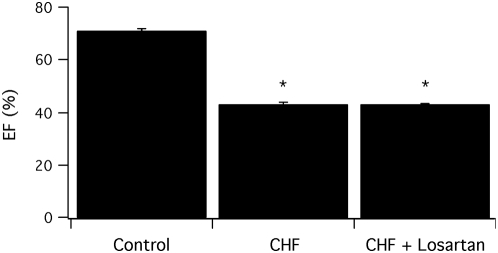
Left ventricular function is reduced in rats following LAD ligation. Bar graph for LVEF (mean±SEM) in uninfarcted, control rats (n = 9), as well as rats 4 weeks following LAD ligation treated with vehicle (CHF, n = 12) or losartan (CHF+losaratan, n = 9). The * indicates a significant difference (p<0.05) compared to control.

The uninfarcted control rats had normal cardiac function with LVEF averaging 71±1% (n = 9). Following LAD ligation, left ventricular function was significantly reduced with an LVEF of 43±1% (n = 12, p<0.05, Fig 1). Losartan therapy, following LAD ligation, did not improve LVEF compared to vehicle treatment (43±3%, n = 9). Compared to vehicle treatment post infarction, the heart rate was significantly higher in the losartan treated rats (304±6 bpm vs 328±8 bpm, p<0.05). Post LAD ligation LVESD (0.15±0.01 cm vs 0.17±0.02 cm) and LVEDD (0.82±0.05 cm vs 0.86±0.04 cm) were slightly larger in the losartan compared to vehicle therapy group, but these differences were not significant (p>0.05).

### MAPK Signaling

**Figure 2:**
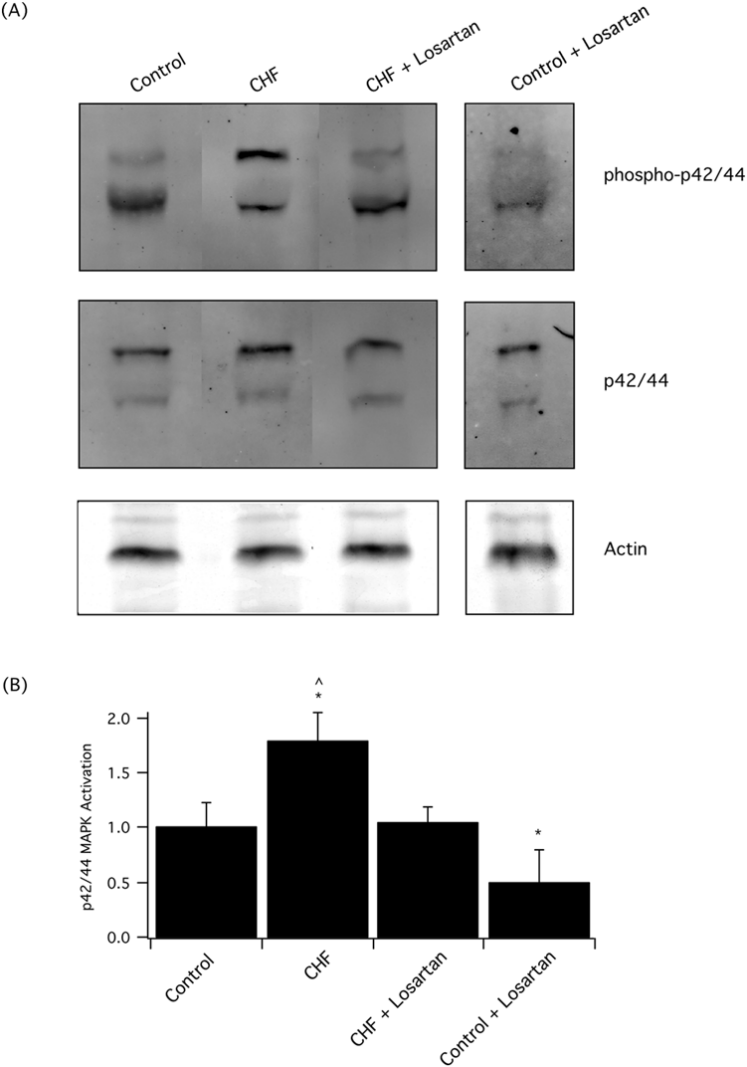
There is a significant activation of p42/44 MAPK following LAD ligation, which is preserved with ARB. (A) Western blots demonstrate that while the expression of p42/44 MAPK does not change following LAD ligation, there is a significant increase in the phosphorylation (activation) of p42/44 MAPK following LAD ligation in the animals treated with vehicle (CHF), which returns to control levels with losartan therapy (CHF+losartan). In addition, treatment of control animals with losartan decreased the activation of p42/44 MAPK. The Coomassie stained gel shows actin, the loading control. (B) Bar graph represents the relative phosphorylation of p42/44 MAPK in control and animals after LAD ligation. The relative phosphorylation of p42/44 MAPK in the controls was set at 1, and the data represent mean±SEM for control (n = 9), and rats following LAD ligation treated with vehicle (CHF, n = 12) or losartan (CHF+losartan, n = 9), as well as control animal treated with losartan (Control+losartan, n = 4). The * indicates a significant difference compared to controls, while the ^∧^ is a significant difference compared to losartan treatment.

**Figure 3:**
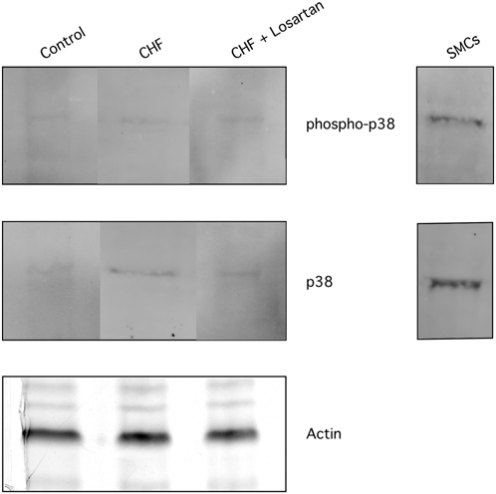
The expression and activation of p38 MAPK does not change following LAD ligation. Western blots with anti-phospho-p38 MAPK antibody and anti-p38 MAPK antibody demonstrate that both p38 MAPK and phospho-p38 MAPK are barely detectable in aortic smooth muscle in control and rats following LAD ligation (CHF, CHF+losartan). Both p38 MAPK and phosphorylated p38 MAPK are detected in cultured rat SMCs. The Coomassie stained gel shows actin, the loading control.

In control rats, as well as in the animals post-LAD ligation, both with and without losartan therapy, p42/44 MAPK, and the activated form of p42/44 MAPK (phospho-p42/44 MAPK) were easily detected (Fig 2). Total p42/44 MAPK expression, normalized to actin, did not change following LAD ligation or with treatment (p>0.05). Four weeks following LAD ligation, there was a significant (p<0.05) activation, or phosphorylation, of p42/44 MAPK in the vehicle treated animals (Fig 2). Compared to vehicle treated rats, losartan therapy following LAD ligation resulted in a significant reduction in the level of activation of p42/44 MAPK (p<0.05), and the relative level of phosphorylation of p42/44 MAPK was not different than that in the uninfarcted control animals (p>0.05, Fig 3).

In contrast to p42/44 MAPK, the expression of p38 MAPK was low; i.e., barely detectable (Fig 3). Similar to total p38 MAPK expression, phospho-p38 MAPK expression was also difficult to detect. There was no change in the intensity of p38 MAPK and phospho-p38 MAPK in control rats or rats following LAD ligation (Fig 3). Due to the low levels of p38 expression, we do not feel that the calculation of relative phosphorylation of p38 MAPK is reliable.

To determine the effect of ARB therapy on MAPK signaling in normal animals, we treated uninfarcted control rats with losartan for four weeks. In control animals, losartan decreased the phosphorylation of p42/44 MAPK compared to vehicle treated animals (1.0±0.2, n = 9 vs. 0.5±0.3, n = 4, p<0.05, Fig 2). However, p38 MAPK expression and activation remained low/undectable in uninfarcted animals treated with losartan.

### MYPT1 Expression

**Figure 4:**
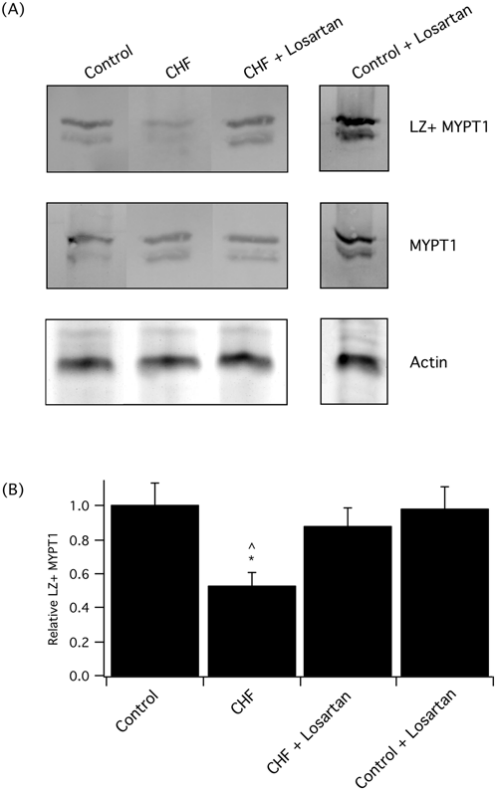
Relative LZ+ MYPT1 expression falls following LAD ligation, which is preserved with ARB. (A) Western blots demonstrate that the relative expression of the LZ+ MYPT1 isoform falls following LAD ligation, but is preserved at control levels in the rats treated with losartan. The Coomassie stained gel shows actin, the loading control. (B) Bar graph represents the relative expression of the LZ+ MYPT1 isoform (given as LZ+ MYPT1/MYPT1) in control rats and animals after LAD ligation. The relative expression of LZ+ MYPT1 in the controls was set at 1. The data represent mean±SEM for control (n = 9), rats following LAD ligation treated with either vehicle (CHF, n = 12) or losartan (CHF+losartan, n = 9), and control animals treated with losartan (Control+losartan, n = 4). The * indicated a significant difference compared to controls, while the ^∧^ is a significant difference compared to losartan treatment.

To determine if LZ+ MYPT1 expression was modulated in heart failure, we used Western blotting with two different antibodies, one of which recognized all MYPT1 isoforms and an anti-LZ+ MYPT1 antibody. Following LAD ligation, consistent with our previous results [22], [24], the relative expression of the LZ+ MYPT1 isoform decreased (p<0.05, Fig 4). However in the losartan treatment group, MYPT1 LZ+ isoform expression remained at control levels (Fig 4). Treatment of control, uninfarcted rats with losartan for 4 weeks did not change the relative expression of the LZ+ MYPT1 isoform (Fig 4, p>0.05). In aortic smooth muscle of normal rats, we have demonstrated that the MYPT1 transcript is 94±6% LZ+ and the expression of MYPT1 is nearly exclusively LZ+ [24], [25]. Thus, it is unlikely that losartan treatment would produce a significant increase in LZ+ MYPT1 expression in the normal rat aorta.

## Discussion

Both ACE-inhibition and ARB therapy post myocardial infarction have been demonstrated in both animals [26], [27], [28], [29], [30] and humans [31], [32] to prolong survival. In our study, LAD ligation produced a moderate reduction in LVEF (LVEF∼40%, Fig 1) and losartan did not improve LVEF. These results are consistent with those of Pourdjabber *et al*. [33]. These investigators demonstrated that both low (3 mg/kg/day) and high (30 mg/kg/day) dose losartan therapy improved post-MI survival. However, the effect on LV function was dependent on infarct size; for large infarcts (LVEF∼10%) both high and low dose losartan treatment improved LV function, whereas neither dose, similar to our results, improved LVEF for moderate (LVEF∼30%) sized infarcts.

During cGMP stimulation, several groups have demonstrated that MYPT1 is phosphorylated at Ser695 [34], [35]. However, there is ample experimental evidence that demonstrates a Ser695 phosphorylation of MYPT1 cannot be the sole mechanism by which cGMP stimulation mediates MLC_20_ dephosphorylation [36]. MYPT1 phosphorylation at Ser695 does not increase MLC phosphatase activity [35], but rather decreases the level of Thr696 phosphorylation to disinhibit the MLC phosphatase and return phosphatase activity to baseline levels [34], [35]. Ca^2+^ desensitization (MLC_20_ dephosphorylation at a constant [Ca^2+^]) occurs during all types of smooth muscle activation, including those that MYPT1 is not phosphorylated at Thr696 [36], [37], [38], [39]. These results contrast with those for the relationship between LZ+ MYPT1 and sensitivity to cGMP; results consistently demonstrate that LZ+ MYPT1 expression determines the sensitivity to cGMP-mediated vasodilatation [13], [18]–[20], [23], [26].

Mendelsoln's group [40] has demonstrated the importance of PKGIα-MYPT1 signaling for vascular function. These investigators generated transgenic mice expressing a mutant PKGIα that was unable to interact with MYPT1, and compared to WT controls, the transgenic mice were both hypertensive and the vascular smooth muscle was less sensitive to both ACh and cGMP mediated vasodilatation. These results demonstrate that normal sensitivity to NO mediated and/or flow mediated vasodilatation is required for the maintenance of normal vascular resistance and blood pressure. Thus a decrease in LZ+ MYPT1 expression would decrease PKGIα-MYPT1 signaling and result in a decrease in sensitivity to NO mediated vasodilatation. We have demonstrated that changes only in LZ+ MYPT1 expression cause changes in the sensitivity to cGMP [21], and a number of groups have demonstrated that the relative expression of the LZ+ MYPT1 isoform determines the sensitivity to cGMP mediated smooth muscle cells relaxation in normal animals, during development and in disease states [13], [18]–[20], [23], [26].

The results of the present study demonstrate that losartan therapy following an MI maintains MYPT1 LZ+ expression at baseline control levels (Fig 4). These results suggest that the ability of ACE-inhibition to maintain LZ+ MYPT1 expression and the normal sensitivity to cGMP mediated smooth muscle relaxation following an MI [22] is not secondary to a decrease in blood pressure, as LZ+ MYPT1 expression is not preserved with prazosin [22], nor an effect of bradykinin, but rather due to the ability of the ACE-inhibitor to decrease Ang II. In heart failure, treatment with an ARB has been demonstrated to preserve the normal sensitivity to ACh induced vasodilatation [41]. Post LAD ligation, we have previously demonstrated that there is no change in the expression of PKGIα [24]. Thus during the treatment of heart failure, these results are consistent with both ACE-inhibitor and ARB therapy decreasing Ang II mediated signaling to alter the vascular phenotype by preventing the fall in LZ+ MYPT1 expression. The maintenance of LZ+ MYPT1 expression would preserve the normal sensitivity to NO mediated vasodilatation.

In animal models of heart failure, activation of MAPK signaling has been demonstrated in cardiac muscle and treatment with ARBs reduces the activation of MAPK signaling pathways [42], [43]. Additionally, the importance of MAPK signaling for the activation of hypertrophic pathways in cultured smooth muscle cells has been well described [44]. However, we are unaware of any data on MAPK expression and/or activation in vascular smooth muscle during heart failure. Nonetheless, our results show that in the normal rat, the expression of p38 MAPK is low and does not change following a LAD ligation or with losartan therapy (Fig 3). Thus despite the well documented importance of p38 MAPK signaling in regulating hypertrophy of cultured smooth muscle cells, p38 MAPK does not appear to be part of a physiologically relevant signaling pathway in rat aortic smooth muscle. In contrast, p42/44 expression is easily detected (Fig 2). Following LAD ligation, there is a significant activation of p42/44 MAPK and losartan decreases the phosphorylation of p42/44 MAPK to control levels (Fig 2). These results show that treatment with an ARB alters the vascular phenotype to preserve normal LZ+ expression and decreases the activation of p42/44 MAPK. Coupled with our previous results [22], which demonstrated that treatment with the ACE inhibitor, captopril, preserves normal LZ+ expression and sensitivity to cGMP mediated vasodilatation, these data suggest that a decrease in Ang II induced activation of the AT1 receptor regulates LZ+ MYPT1 expression, possibly by decreasing p42/44 MAPK signaling. During the activation of B and T lymphocytes, alternative mRNA splicing of the cell surface marker CD44 has been demonstrated to depend on activation of Raf1-MEK-p42/44 MAPK signaling, but not p38 MAPK [45], which suggests that p42/44 MAPK signaling could regulate alternative splicing of the MYPT1 transcript. Further recent evidence suggests that Tra2β is an important regulator of alternative splicing of the 31 bp 3′ exon (MYPT1 E23), which codes for the presence or absence of the MYPT1 LZ [46]. These investigators demonstrated that Tra2β binds to the MYPT1 E23 sequence and transactivates splicing of MYPT1 E23 both *in vitro* and *in vivo*. Although the pathway between activation of the AT1 receptor and LZ+ MYPT1 expression has yet to be elucidated, the p42/44 MAPK signaling pathway has been demonstrated to have a role in alternative mRNA splicing [45], and could regulate LZ+ MYPT1 expression, possibly by controlling the expression of Tra2β.

Our results demonstrate that Ang II stimulation of AT1 receptor both activates p42/44 MAPK signaling and decreases the expression of the LZ+ MYPT1 isoform. Thus the ability of ACE-inhibition and ARBs to counter this deleterious effect on the vascular phenotype could explain their beneficial effect, compared to other agents that decrease afterload, on morbidity and mortality in the treatment of heart failure. Further, our data may suggest that blocking the p42/44 MAPK cascade could preserve normal vascular reactivity and reverse the vascular dysfunction associated with heart failure.

## Materials and Methods

### Ethics Statement

All work was performed in accordance with the APS principles for ethical treatment of animals, using a protocol approved by the IACUC of the Mayo Medical School.

### MI surgery

Myocardial infarction was induced by LAD ligation as previously reported [22]. Briefly using a protocol approved by the IACUC of the Mayo Medical School, adult male Sprague-Dawley rats (Harlan,IN) weighing 300–400 g were anesthesized with an intramuscular injection of a 5∶1 mixture of ketamine (100 mg/mL) and xylazine (100 mg/mL). After intubation, rats were mechanically ventilated using a small animal ventilator (model 683, Harvard). A left lateral thoracotomy was performed along the 5^th^ intercostal space, followed by dissection of pericardium to expose the heart. The left anterior descending coronary artery (LAD) was visualized and ligated using 6-0 prolene suture (Ethicon). After chest closure with running 3-0 vicryl suture (Ethicon), the air in the chest cavity was evacuated, and the rats were then placed in a warmed recovery chamber overnight. Control animals underwent a sham procedure, where the only difference was that the LAD was not ligated. Following surgery, animals were divided into a vehicle treated or losartan treated group. For the losartan group, rats were allowed to drink water containing 50 mg/l losartan, *ad libitum*, which is approximately 3 mg/kg/day for 4 weeks.

### Echocardiograms

Prior to imaging, animals were anesthetized with 2% isoflurane. The anterior chest hair was removed, and the rats were placed in supine position and the EKG was continuously monitored [22], [24], [47]. The echocardiographic studies were performed using the Vivid 7 ultrasound system (GE Medical Systems, Milwaukee, Wis) and a 10S transducer (11.5 MHz) with high temporal and spatial resolution. Grey scale images were recorded in the parasternal long and short axis views. M-mode tracings were obtained at the level of the papillary muscles at a speed of 200 mm/sec. Measurements were performed offline using the EchoPAC PC software (GE Medical Systems, version 3.1.3). Fractional shortening was obtained using the formula: ((LVIDd−LVIDs)/LVDd)×100 where LVIDd is the LV diameter at end-diastole and LVIDs is the LV diameter at end-systole and the LVEF was calculated as ((LV end-diastolic volume−LV end-systolic volume)/(LV end-diastolic volume))×100%.

### Western Blots

The aorta was dissected and cleaned of connective tissue, and protein extracted in SDS sample buffer. The sample was resolved by SDS-polyacrylamide gel electrophoresis (SDS-PAGE) using 8% (29∶1) gels for MYPT1 and 12% (29∶1) gels for MAPK. The actin band on Coomassie stained gels was used to normalize protein loading. Following SDS-PAGE separation, protein bands were transferred to a nitrocellulose membrane in buffer containing 1× NuPAGE transfer buffer, 20% methanol, and then blocked in 5% nonfat dry milk with 1×TBS including 0.05% (v/v) TWEEN for 1 hour. Antibodies used were a polyclonal anti-MYPT1 (Covance F38.130, PRB-457C), a monoclonal anti-LZ+ MYPT1 isoform [48], anti-p38 MAPK, anti-p42/44 MAPK, anti-phospho-p38 MAPK and anti-phospho-p42/44 MAPK (Cell Signaling #9212, #9102, #9211S, #9101S, respectively). Alkaline phosphatase was used to detect MYPT1, whereas enhanced chemiluminescence (ECL) was used for the detection of MAPK, and the identified protein bands were visualized using a Typhoon 9400 Variable Mode Imager (Amersham Biosciences). Protein expression was first normalized for the actin intensity on the corresponding Coomassie stained SDS. Then, the relative activation of MAPK was calculated as the normalized intensity of the band(s) representing phospho-p38 MAPK or phospho-p42/44 MAPK divided by the normalized intensity of the band corresponding nonphosphorylated protein, and the level in the controls was set as 1. Similarly, the relative expression of the LZ+ MYPT1 isoform was calculated as the intensity of the band(s) representing the LZ+ MYPT1 isoform divided by the intensity of the band(s) representing total MYPT1, and the control level was set to 1.

### Statistical Analysis

Data in the text are given as mean±SEM, and differences between groups were determined using an ANOVA with significance at the p<0.05 level.
